# Freeze–Thaw Damage Mechanism Analysis of SBS Asphalt Mixture Containing Basalt Fiber and Lignocellulosic Fiber Based on Microscopic Void Characteristics

**DOI:** 10.3390/polym15193887

**Published:** 2023-09-26

**Authors:** Wensheng Wang, Liansheng Yang, Honghai Cui, Fei Wu, Yongchun Cheng, Chunyu Liang

**Affiliations:** 1 College of Transportation, Jilin University, Changchun 130025, China; yangls1719@mails.jlu.edu.cn (L.Y.); wufei1719@mails.jlu.edu.cn (F.W.); chengyc@jlu.edu.cn (Y.C.); 2 Jilin Traffic Planning and Design Institute, Changchun 130021, China; cuihh23@outlook.com

**Keywords:** SBS asphalt, basalt fiber, lignocellulosic fiber, void characteristics, freeze–thaw damage

## Abstract

Freeze–thaw effects pose the significant challenge to asphalt pavement durability, leading to various types of distress and deterioration. This study investigates the freeze–thaw damage mechanism of Styrene–Butadiene–Styrene (SBS) asphalt mixtures containing reinforcement fibers, specifically basalt fiber as well as lignocellulosic fiber, through a microscopic void characteristics analysis. This investigation aims to understand how the presence of basalt fiber as well as lignocellulosic fiber influences void characteristics for SBS asphalt mixtures during freeze–thaw cycles. A comprehensive experimental program was conducted for the void and mechanical characteristics, which involved the preparation of SBS asphalt mixtures containing basalt fiber as well as lignocellulosic fiber. The mechanical performances of the two types of asphalt mixtures decrease with more freeze–thaw cycles. The decline is faster initially and gradually slows down. Basalt-fiber-modified SMA-13 has higher air void content and mechanical properties compared to lignocellulosic-fiber-modified SMA-13, indicating that adding basalt fibers improves the mechanical performances of SMA-13 asphalt mixture. Both types of asphalt mixtures experience increasing damage with more freeze–thaw cycles, indicating irreversible damage. The stability damage levels are similar, but basalt-fiber-modified SMA-13 has lower splitting strength damage and stiffness modulus damage compared to lignocellulosic-fiber-modified SMA-13. This suggests that adding basalt fibers enhances the resistance to freeze–thaw damage. Surface wear of asphalt mixtures under repeated freeze–thaw cycles is a complex and dynamic process. Fractal theory can uncover the mechanism of surface wear, while describing surface wear behavior and void deformation characteristics using fractal dimension, angularity, roundness, and aspect ratio is a logical and effective approach. The findings provide insights into freeze–thaw damage mechanisms at the microscopic level, highlighting the effects of reinforcement fibers. They provide valuable insights that can be used to optimize the design and maintenance of asphalt pavements.

## 1. Introduction

Freeze–thaw cycles pose a significant challenge to asphalt pavements, leading to various types of distress and deterioration [1,2]. Particularly, asphalt mixtures containing SBS bituminous binders have been widely used due to their improved rutting resistance and durability [3,4]. However, the freeze–thaw damage mechanisms in SBS asphalt mixtures incorporating reinforcement fibers including natural fibers and prior composites, such as basalt fiber and lignocellulosic fiber, are not yet fully understood [5,6,7,8,9]. Understanding the freeze–thaw damage mechanisms is essential to develop effective strategies for mitigating such distress as well as enhancing the long-term performance of asphalt pavement.

Freeze–thaw cycles, water action, and traffic are the main distresses of asphalt pavements in seasonal frozen regions [10,11]. These factors can lead to the deterioration of the pavement, causing cracks, rutting, and other forms of damage. To mitigate these issues, researchers have explored the use of various additives and reinforcements in asphalt mixtures [12,13]. In recent years, the incorporation of fibers into asphalt mixtures has gained considerable attention as a potential solution to enhance the material’s resistance to freeze–thaw damage. Among the various types of fibers, basalt fibers and lignocellulosic fibers have shown promising results in improving the mechanical properties and durability of asphalt mixtures [14,15,16]. Lignocellulosic fibers are derived from plant-based materials and offer advantages, such as natural abundance, low cost, and renewable nature. Lignocellulosic fiber has also been considered as a potential additive in asphalt mixtures due to its reinforcement properties and environmental benefits. This fiber can improve the toughness and durability of the mixture, as well as enhance its resistance to cracking and rutting [17,18]. Additionally, basalt fibers derived from rocks possess excellent mechanical strength and high-temperature resistance [19]. The addition of basalt fiber has shown promise in improving mechanical properties in frozen regions. Previous studies have indicated that increasing the basalt fiber content initially enhances the pavement performance, including the freeze–thaw resistance [20]. However, further investigation is needed to compare the effects of basalt fiber and lignocellulosic fiber and their long-term effects on the mixture’s mechanical properties.

Microscopic void characteristics play a crucial role in the freeze–thaw damage mechanism for bituminous mixtures [21,22,23]. When an asphalt mixture is exposed to freeze–thaw cycles, the presence of voids within the mixture becomes a pathway for water ingress. Water can penetrate into these voids, leading to the formation of ice crystals during freezing. The expansion of these ice crystals exerts pressure on the surrounding materials, causing internal stresses and potentially leading to various forms of damage, such as cracking and debonding [24,25]. The size, shape, and distribution of voids within the asphalt matrix significantly influence the susceptibility of the mixture to freeze–thaw damage [26]. Larger voids provide more space for water to accumulate and freeze, resulting in higher levels of stress and damage. Irregularly shaped voids can create stress concentration points, making the mixture more prone to cracking. Moreover, the distribution of voids affects the connectivity of water pathways, influencing the extent of water ingress and the subsequent damage [27,28]. In this context, it is important to understand the influence of basalt fibers as well as lignocellulosic fibers on microscopic void characteristics of SBS bituminous mixtures. The joining of these fibers into the bituminous mixture aims to enhance its resistance to freeze–thaw damage. By incorporating basalt fibers and lignocellulosic fibers into the SBS asphalt mixture, the characteristics of the microscopic voids can be altered [29,30]. The fibers can act as a filler, occupying some of the void spaces and reducing the overall porosity of the mixture. This, in turn, can inhibit water ingress and the formation of ice crystals [31]. Additionally, the presence of fibers can promote better interlocking and bonding between the asphalt matrix and the aggregates, enhancing the overall integrity of the mixture and reducing the potential for damage [32]. Therefore, a comprehensive understanding of the effect of basalt fibers and lignocellulosic fibers on the microscopic void characteristics of SBS asphalt mixtures is essential for evaluating their effectiveness in mitigating freeze–thaw damage. Through experimental investigations and microscopic analysis techniques, the size, shape, and distribution of voids can be quantified and compared between mixtures with and without fiber additions. This analysis will provide insights into how the fibers modify the interior structure and contribute to the resistance against freeze–thaw damage.

In view of the vital role of microscopic void characteristics in evaluating freeze–thaw resistance for bituminous mixtures, this study focuses on analyzing the freeze–thaw damage mechanism of SBS asphalt mixtures containing basalt fiber and lignocellulosic fiber based on the microscopic void characteristics. The primary objective of this research is to investigate how basalt fiber and lignocellulosic fiber affect the microstructure and void features for SBS bituminous mixtures during freeze–thaw cycles. Furthermore, this study aims to identify the key factors influencing the freeze–thaw damage mechanisms and quantify their influences on the performance of bituminous pavements. The findings could have important implications for the design and construction of asphalt pavements and can be useful for engineers and designers involved in asphalt pavement design, as they provide a potential solution to the problem of freeze–thaw damage in asphalt pavements. The microscopic void characteristics analysis could provide a valuable understanding of the intricate mechanisms underlying the freeze–thaw damage processes, allowing for the development of more effective mitigation strategies.

## 2. Materials and Methods

### 2.1. Raw Materilas

This research used modified bitumen SBS type I-C provided by Jilin Zhonghai Asphalt Co., Ltd. (Yingkou, China), which appears as black and semi-solid. Referring to JTG E20-2011 [33], the technical parameters, such as the penetration grade as well as the softening point of bitumen, were tested. The experimental results meet the technical standard, and the specific testing indicators are shown in Table 1.

The composition structure of the Stone Matrix Asphalt (SMA) mixture belongs to a dense-skeleton structure, where mineral aggregates play the role of skeleton and filling, greatly influencing the overall performance of the SMA mixture. The coarse aggregate used in this study is basalt gravel produced in Liaoyuan, which is clean and free from impurities. The fine aggregate is manufactured sand, which is clean and free from impurities. The mineral powder used is dry and free from agglomeration. Referring to the testing methods specified in JTG E20-2011, the basic parameters of the raw materials, including coarse aggregate, fine aggregate, and mineral powder, were tested. The experimental results are shown in Table 2, Table 3 and Table 4.

Fiber stabilizers in SMA mixtures play a role in adsorbing asphalt and improving toughness [34,35]. Common fiber stabilizers used in SMA mixtures include lignocellulosic fibers, polyester fibers, and mineral fibers. Among them, lignocellulosic fibers are widely used. In this study, basalt fibers were used to replace lignocellulosic fibers. The performance indicators of the two fibers were determined, and experimental tests were conducted on SMA mixtures with both fibers to comprehensively evaluate them. The used lignocellulosic fiber was produced by Jilin Mingsheng Technology Co., Ltd. (Changchun, China). After high-temperature and chemical treatments, and the appearance of type flocculent lignin fiber is gray and fluffy. The basalt fiber was produced by Jilin Tongxin Basalt Fiber Co., Ltd. (Tonghua, China) after melt spinning and heat treatments, and the appearance of type basalt fiber short cut fiber is dark brown and needle-like. The experimental results for two fibers are summarized in Table 5. The two types of fiber materials have different properties and play different roles in asphalt mixtures, so their dosage in asphalt mixtures is also different. At a higher fiber dosage, the dispersion cause of the fiber is poor, which can easily lead to an agglomeration phenomenon and insufficient dispersion in the asphalt mixture, resulting in a poor mixing effect. When the fiber dosage is too low, it cannot adsorb bitumen or enhance the toughness of the bituminous mixture, leading to a reduction in the mechanical performance. In order to give the experiment certain engineering significance, the dosage of lignocellulosic fiber was determined as 0.3%. According to the relevant literature on basalt fiber, the corresponding dosage was set at 0.4% [20].

### 2.2. Asphalt Mixture Proportion Design

**Figure 1:**
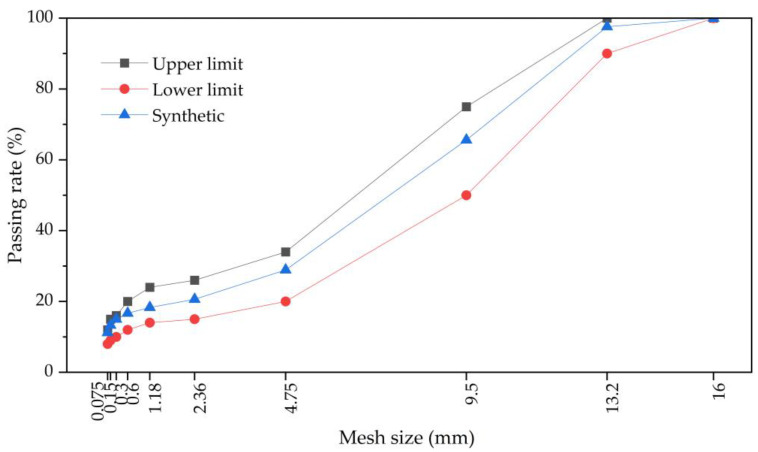
SMA-13 asphalt mixture synthetic grading curve.

This study takes the seasonal frozen climate as the research background, and the asphalt mixture used for pavement construction needs to have good performance in terms of low-temperature crack resistance, durability, etc. The SMA mixture is a dense-skeletal structure that combines the advantages of AC as well as OGFC structures. It has superior wear resistance, low-temperature crack resistance, and it is suitable for high-grade pavement and heavily trafficked road sections. Based on the asphalt surface layer design of the actual expressway test section, the final type of asphalt mixture used was SMA-13. The gradation design is plotted in Figure 1.

In order to compare basalt-fiber-modified SMA-13 with the lignocellulosic-fiber-modified SMA-13 mixture, the two asphalt mixtures in this study used the same gradation. Then, it was necessary to determine the most suitable asphalt-to-aggregate ratio. If the ratio is too high, it will aggravate the phenomenon of asphalt mixture segregation, and it is prone to bleeding in high-temperature summer environments and under traffic loads, seriously affecting the pavement. If the asphalt-to-aggregate ratio is too low, it cannot ensure the cohesion between aggregates, resulting in reduced durability and stability. The fibers used were basalt fiber and lignocellulosic fiber, and the two fibers have different oil absorption rates. Lignocellulosic fiber has a stronger ability to adsorb asphalt than basalt fiber. Therefore, when forming the specimens, the lignocellulosic-fiber-modified asphalt mixture requires a higher asphalt content compared to the basalt-fiber-modified bituminous mixture. In the mix design, the asphalt-to-aggregate ratio needs to be calculated separately for the two asphalt mixtures. According to engineering experience, the ratio for the basalt-fiber-modified SMA-13 mixture should be 0.3% to 0.5% lower than the lignocellulosic-fiber-modified SMA-13 mixture. Therefore, an initial value of 5.9% was chosen for the basalt-fiber-modified SMA-13 mixture, and a value of 6.3% was chosen for the lignocellulosic-fiber-modified SMA-13 mixture. The asphalt-to-aggregate ratios of both mixtures were then adjusted up and down by 0.2%. Marshall specimens were formed for the six groups using the Marshall compaction method, and the specimens were tested. The experimental results are listed in Table 6.

Regarding the void content design of the SMA, due to its characteristics of skeletal structure and higher asphalt-to-aggregate ratio, an excessively high void content will lead to a reduction in structural stability as well as durability for the SMA. On the other hand, a too low void content will cause bleeding on the pavement surface and the formation of ruts. According to Chinese regulation “Technical Specifications for Construction of Highway Asphalt Pavement,” the void content range is 3~4%. Therefore, a median void content of 3.5% was set. Under this void content, both types of asphalt mixtures met the specifications and showed good performance in various indicators. Finally, the ratio for the basalt-fiber-modified SMA-13 mixture was determined to be 5.9%, and the asphalt-to-aggregate ratio for the lignocellulosic-fiber-modified SMA-13 mixture was set at 6.3%.

### 2.3. Experimental Methods

The specified freeze–thaw cycle test involved vacuum saturating the test specimens with water, quickly removing them from the water and placing them in bags, injecting a certain amount of water into the bags and removing the air, sealing the bags, and freezing them for 16 h at −18 °C in a low-temperature chamber. After that, the specimens were taken out and placed in a 60 °C water bath for 8 h. This completed one freeze–thaw cycle, and a total of 15 freeze–thaw cycles were conducted. To increase the experimental precision, 4 specimens were selected for parallel tests. Considering the rapid decline in mechanical properties for the bituminous mixture during the early freeze–thaw period and their gradual stabilization later on, the tests were intensified in the early stage. Therefore, tests were conducted on the asphalt mixture before undergoing 0~15 cycles.

According to testing methods specified in the JTG E20-2011 code and the technical guidelines, the bulk density test method was selected to test the two types of bituminous mixtures after freeze–thaw cycles. In accordance with the Marshall stability test method specified in the JTG E20-2011 code, the height of the specimens during molding had a significant impact on the test results, and strict control over the height was required during molding. Specimens that did not meet the requirements were discarded, and an asphalt mixture stability tester was used for testing. According to the freeze–thaw splitting test method for asphalt mixtures specified in the JTG E20-2011 code, the asphalt mixtures containing two types of fibers were subjected to splitting tests after different freeze–thaw cycles. The testing method for the indirect tensile test modulus of elasticity referred to the existing literature [36], and it was used to test the asphalt mixtures containing two types of fibers after different freeze–thaw cycles.

Starting from CT scanning technology, the CT imaging quality was improved by selecting appropriate scanning parameters and adjusting the fixation of the scanning specimens. The image quality was further enhanced through image adjustment and processing, ultimately improving the precision of the research results. The selection of relevant parameters and the introduction of technical principles can be found in the existing literature [23]. The final determination of the main CT scanning parameters was a scan thickness of 0.6 mm, a tube voltage of 130 kV, and a milliamperes-second of 250 mAs. Through specimen fixation, the adjustment of the window width and the window level, etc., the CT imaging was more suitable for subsequent image processing and information extraction. Based on digital image processing technology, image denoising, image enhancement, and image segmentation, methods were used to make the image details clearer for subsequent parameter extraction and analysis.

## 3. Results and Discussion

### 3.1. Damage Characteristics of Fiber-Modified SMA Mixture under Freeze–Thaw Cycles

#### 3.1.1. Void Content Analysis

**Figure 2:**
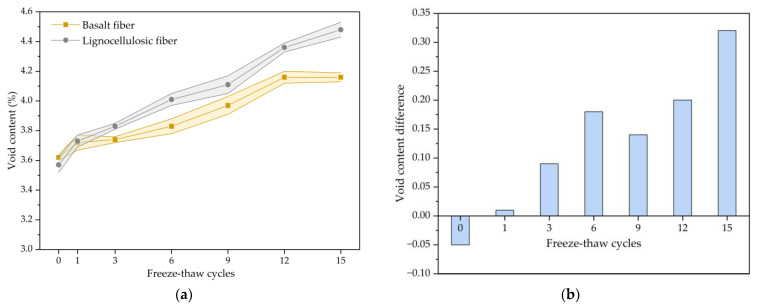
The void content results of fiber-modified SMA mixture: (**a**) Change trend of void content; (**b**) Void content difference between lignocellulosic-fiber- and basalt-fiber-modified SMA.

Void content testing was conducted on specimens subjected to varying quantities of freezing and thawing repeated processes, and the experimental results were obtained according to JTG E20-2011. The calculated results can be found in Figure 2a. From Figure 2a, it can be observed that void content curves for both types of bituminous mixtures were similar, showing an upward trend with an increase in freezing and thawing repeated processes. The void content of the basalt-fiber-modified asphalt mixture increased by 14.9% after 15 cycles, while that of the lignocellulosic-fiber-modified asphalt mixture increased by 25.5%. The void content showed significant changes in the early period for freezing and thawing repeated processes but tended to stabilize as the freezing and thawing repeated process continued. The initial void content of the basalt-fiber-modified asphalt mixture was slightly larger than that of the lignocellulosic-fiber-modified asphalt mixture, but the increase in the void content was slower, and after the first freeze–thaw cycle, it remained lower than that of the lignin-fiber-modified asphalt mixture. The reason for this phenomenon may be that the length and modulus of lignocellulosic fiber are smaller than those of basalt fiber, which have weaker resistance to deformation during freeze–thaw processes, resulting in higher porosity.

According to Figure 2b, the difference in the void content between two types of bituminous mixtures is −0.05% before freeze–thaw cycling. As the freezing and thawing repeated process progressed, the difference in the void content showed an increasing trend and reached 0.32% after 15 cycles. The basalt-fiber-modified asphalt mixture initially had a higher void content, but the rate of the void content that increased during the freezing and thawing repeated process was lower than that of the lignocellulosic-fiber-modified bituminous mixture. This indicates that adding basalt fibers improves the structural stability and deformation resistance of bituminous mixture. As the freezing and thawing repeated process continued, the advantages of basalt fibers in resisting freeze–thaw damage gradually became evident.

Asphalt mixture is a composite material, and there is initial damage during the Marshall specimen molding. The void content is one of the indicators of initial damage. During the freezing and thawing repeated process, the temperature during the low-temperature freezing process is −18 °C, and the internal void water of the specimen freezes and expands at low temperatures, exerting freeze–thaw pressure on the internal structure of the specimen. During the melting process, the water bath temperature is 60 °C and the adhesion between the asphalt and the aggregate deteriorates, causing some of the asphalt to peel off from the specimen under the combined action of the water and temperature. Under this freeze–thaw damage, the internal structure of the specimen deforms and eventually fails based on the initial damage. This process is irreversible, so the void content of both types of asphalt mixtures increases monotonously based on the initial void content. The development of internal voids in the asphalt mixture during a freeze–thaw cycle can be divided into three stages: i.e., (I) the formation of new, tiny voids; (II) the deformation of voids containing water under the freeze–thaw pressure of ice; and (III) the thinning of old adjacent voids during deformation and the merging into larger voids. In this experiment, the internal structure of the asphalt mixture undergoes significant deformation in the early stage of freeze–thaw. As the freeze–thaw cycle progresses, the formation of new voids gradually reaches a dynamic equilibrium with the merging of voids, and the internal structure stabilizes, resulting in a decrease in the deformation rate and a relatively smaller change in the void content later on.

The elastic modulus of basalt fiber is higher than that of lignocellulosic fiber, making it more difficult to compact the basalt-fiber-modified asphalt mixture during molding, resulting in a higher void content than the lignocellulosic-fiber-modified asphalt mixture. To ensure the required void content of the specimen, a larger compaction effort is needed, and the characteristic of basalt fiber provides a theoretical basis for increasing the number of compaction passes during the laying of experimental road sections. The basalt fiber is coated with a coupling agent, which improves the dispersibility of the fiber bundles in the asphalt mixture and enhances the bonding effect with the asphalt. The basalt fibers are randomly distributed in the asphalt mixture, forming a net-like structure, which improves the structural stability of the asphalt mixture. The small diameter and large specific surface area of the basalt fiber enable it to fully contact and adsorb the asphalt. Therefore, the addition of basalt fiber in the asphalt mixture effectively prevents the deformation of the mixture structure. On a macroscopic level, the basalt-fiber-modified asphalt mixture has a lower void content and slower growth rate in the freeze–thaw cycle compared to the lignocellulosic-fiber-modified asphalt mixture.

#### 3.1.2. Marshall Stability Analysis

**Figure 3:**
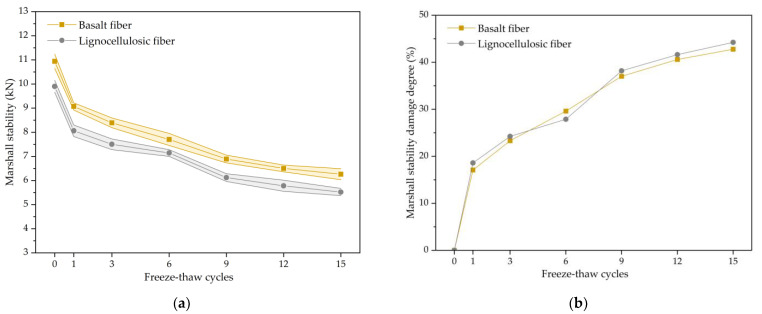
The Mashall stability results of fiber-modified SMA mixture: (**a**) Change trend of Mashall stability; (**b**) Change trend of Mashall stability damage degree.

Marshall stability tests were conducted on specimens subjected to different freezing and thawing repeated processes. The variation in the Marshall stability with the cycle number is shown in Figure 3a, while the change trend in the Marshall stability damage degree with freeze–thaw cycles is depicted in Figure 3b.

According to Figure 3a, the Marshall stability for both bituminous mixtures decreased as the freeze–thaw cycle increased. At 15 cycles, the Marshall stability for the basalt-fiber-modified bituminous mixture decreased by 42.8%, while the Marshall stability for the lignocellulosic-fiber-modified bituminous mixture decreased by 44.2%. The decrease in the Marshall stability of the asphalt mixtures is mainly due to the damaging effect of freezing and expanding forces generated by water freezing in low-temperature environments. The Marshall stability curve showed a significant initial decline during the early period in the freezing and thawing repeated process, which is attributed to significant deformation and performance deterioration within the asphalt mixture. As the experiment progressed, the internal structure tended to reach dynamic stability, and the decline in performance became less pronounced. In terms of the Marshall stability value, the basalt-fiber-modified asphalt mixture consistently exhibited higher stability than the lignocellulosic-fiber-modified asphalt mixture across all freeze–thaw stages. This is because the basalt fiber had higher tensile strength compared to the lignocellulosic fiber, enabling it to resist structural deformation and improve stability within bituminous mixture. When analyzing air voids, the basalt-fiber-modified bituminous mixture had larger voids and higher stability compared to the lignocellulosic-fiber-modified bituminous mixture before freeze–thaw. This is attributed to the basalt fiber forming a network within the asphalt mixture, which, although it impedes compaction, enhances the structural stability for the bituminous mixture. This indicates that adding basalt fiber would efficiently develop the ability of the bituminous mixture to resist external loads and enhance its resistance against water damage and stripping.

Upon observation and analysis of Figure 3b, it can be seen that the Marshall stability damage of asphalt mixtures, when adding both types of fibers, increased with an increase in the freezing and thawing repeated process. This is because the structure and materials for asphalt mixture deteriorate under the freezing and thawing repeated process, leading to a decrease in the mechanical properties of the specimens. The Marshall stability damage for the asphalt mixture exhibited a rapid initial change during an early period in the freezing and thawing repeated process and gradually stabilized thereafter. This is because significant internal structural changes occur during an early period of damage, leading to the formation of additional microvoids in the material, causing a rapid decline in its mechanical properties. Subsequently, the newly formed voids gradually reach a dynamic equilibrium with the merged voids, leading to the stabilization of the performance. The Marshall stability damage of the bituminous mixture through the addition of basalt fiber is slightly lower compared to that of the mixture with lignocellulosic fiber. This is attributed to the elevated strength of basalt fiber, which is randomly distributed, making the structure of the mixture more stable. This enables it to effectively resist internal structural deformation and reduce the damage to the mixture.

#### 3.1.3. Splitting Tensile Strength Analysis

**Figure 4:**
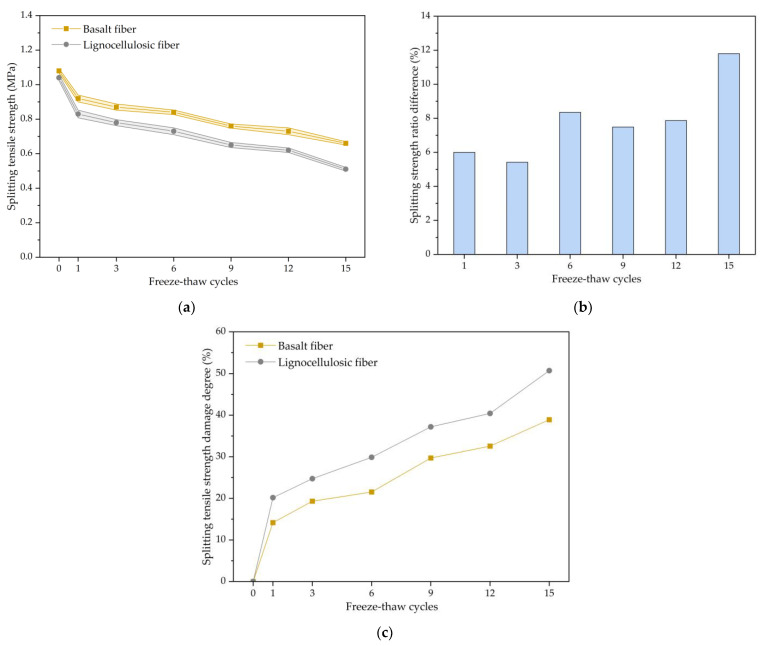
The splitting tensile strength results of fiber-modified SMA mixture: (**a**) Change trend of splitting tensile strength; (**b**) Splitting strength ratio difference between lignocellulosic-fiber- and basalt-fiber-modified SMA; (**c**) Change trend of splitting tensile strength damage degree.

Figure 4a illustrates the disparity in the splitting tensile strength between the two mixtures subjected to varying freezing and thawing repeated processes. By observing and analyzing Figure 4a, it can be seen that both the splitting tensile strengths exhibited a decline in the freezing and thawing repeated process. The decrease was more significant in the early stages of the freezing and thawing repeated process, and the splitting strength tended to stabilize as the test progressed. After 15 cycles, the splitting strength of the basalt-fiber-modified bituminous mixture exhibited a decline of 0.42 MPa, while the splitting strength of the lignocellulosic-fiber-modified asphalt mixture decreased by 0.53 MPa. This is because water enters the mixture and the freezing expansion force generated by ice formation at low temperatures causes the expansion and extension for voids, resulting in internal structural damage to the mixture. Meanwhile, under high-temperature conditions, the bonding strength between the asphalt and the aggregate weakens, causing a decrease in the splitting strength. As the number of freeze–thaw cycles increases, the internal structure of the asphalt mixture tends to stabilize, and the expansion and interconnected voids partially dissipate the freezing expansion force of the ice, resulting in a slower decrease in the splitting strength. The splitting tensile strength of the basalt-fiber-modified asphalt mixture is always higher than that of the lignocellulosic-fiber-modified asphalt mixture. Compared with the lignocellulosic-fiber-modified asphalt mixture, the splitting tensile strength of the basalt-fiber-modified asphalt mixture was increased by 3.8% before any freeze–thaw cycles and by 29.4% after 15 cycles. The experimental results indicate that adding basalt fiber can improve the splitting strength of the mixture and enhance the frost resistance of the asphalt mixture. As the cycles progressed, the improvement effect of the basalt fiber on the asphalt mixture became more prominent. This is because basalt fibers are scattered and randomly distributed inside the asphalt mixture, forming a fiber network that enhances the structural stability of the mixture.

By observing Figure 4b, it can be seen that the splitting strength ratios for both asphalt mixtures decreased monotonically as the freeze–thaw cycles increased. The decrease was more significant in the early stages of the freeze–thaw cycles and tended to flatten as the test progressed, which is consistent with the trend of split strength variation. The splitting strength ratios for the basalt-fiber-modified asphalt mixture are higher than those of lignocellulosic-fiber-modified asphalt mixture at each freeze–thaw stage. The improvement values of the splitting strength ratio were always positive, indicating that adding basalt fiber can effectively enhance the frost resistance of the mixture. The improvement values showed an overall increasing trend, with a 6.0% increase in the splitting strength ratio after the first cycle and an 11.8% increase after the 15th cycle. This demonstrates that the advantages of basalt fiber in frost resistance become more prominent as the freeze–thaw cycle increases.

The variation of damage degree with the number of freeze–thaw cycles can be seen in Figure 4c. The damage degree of the splitting tensile strength of both asphalt mixtures gradually increased with the increase in the freeze–thaw cycles, showing significant changes in the early stages of the freeze–thaw cycles and gradually stabilizing as the test progressed. This is because under the action of freeze–thaw cycles, the voids in the mixture gradually expand and extend, and the structural damage accumulates, leading to a rapid increase in the damage degree. As the test progresses, the internal structure of the mixture reaches a dynamic equilibrium state, and the rate of increase in the damage degree slows down. The damage degree of the basalt-fiber-modified asphalt mixture was lower than that of the lignocellulosic-fiber-modified asphalt mixture at each stage of the freeze–thaw cycles. After 15 cycles, the damage degree of the splitting tensile strength for the basalt-fiber-modified asphalt mixture was 38.91%, while it was 50.71% for the lignocellulosic-fiber-modified asphalt mixture. This indicates that basalt fiber has a good ability to resist freeze–thaw damage, and its resistance to freeze–thaw damage becomes more prominent with the increase in freeze–thaw cycles.

#### 3.1.4. Indirect Tensile Stiffness Modulus Analysis

**Figure 5:**
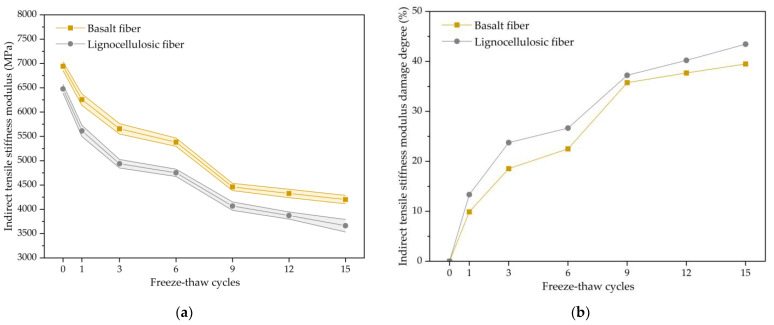
The indirect tensile stiffness modulus results of fiber-modified SMA mixture: (**a**) Change trend of indirect tensile stiffness modulus; (**b**) Change trend of indirect tensile stiffness modulus damage degree.

Experimental specimens in the freezing and thawing repeated process were tested for the indirect tensile stiffness modulus, which showed variations during the freezing and thawing repeated process, as seen in Figure 5a. From Figure 5a, it can be seen that the stiffness modulus for both types of fiber-modified bituminous concrete generally declined monotonically during the freeze–thaw cycle tests. When subjected to 15 cycles, the stiffness modulus for the basalt-fiber-modified bituminous concretes decreased by 2742 MPa, while that of the lignocellulosic-fiber-modified bituminous mixture declined by 2814 MPa. The stiffness modulus showed a larger decrease during the early period for the freezing and thawing repeated process, and the speed of decrease tended to stabilize as the tests progressed. This is because the internal structure experiences significant damage in the early freezing and thawing period, resulting in a substantial decrease in its load resistance capacity. With the progression of the tests, the internal structure gradually reaches a dynamic stable state, leading to a more gradual change in the stiffness modulus. The stiffness modulus of the basalt-fiber-modified bituminous concretes was higher than that of the lignocellulosic-fiber-modified bituminous concretes at all stages of freeze–thaw damage, indicating that adding basalt can enhance low-temperature anti-cracking. Analyzing the void content reveals that, in the unfrozen state, the basalt-fiber-modified bituminous concrete had a larger void content and a higher stiffness modulus compared to the lignocellulosic-fiber-modified asphalt mixture. This suggests that the joining of basalt fiber does not reduce the performance by making it difficult to compact. This is because basalt fiber acts as reinforcement as well as crack resistance. During the freezing and thawing repeated process, the basalt fiber could share part of the stress in the bituminous mixture, thereby enhancing its ability to resist low-temperature cracking.

From Figure 5b, it can be seen that the damage degree of both bituminous mixtures increased monotonically in the freezing and thawing repeated process. After 15 cycles, the damage degree for the basalt-fiber-modified bituminous concrete was 39.5%, while that of the lignocellulosic-fiber-modified asphalt mixture was 43.5%. This study shows that as the tests progressed, the material performance deteriorated and the damage degree increased. The rate of change in the damage degree was faster in the early stages of the test and slowed down as the test progressed. This is because under the freezing and thawing repeated process, the internal structure is damaged, and the adhesion between the asphalt and aggregate decreases. Some fine aggregates are washed away by the water flow, further damaging the internal structure and reducing the mechanical properties. As the test progresses, the structure tends to stabilize, and the increase rate in the damage degree slows down. Throughout all stages of the test, the damage degree curve of the basalt-fiber-modified bituminous concrete was always below that of the lignocellulosic-fiber-modified bituminous concrete. This study indicates that adding basalt fiber could effectively reduce the damage degree of concrete. This is because basalt fiber forms a fiber network inside the concrete, preventing the expansion of internal voids and thereby improving low-temperature anti-cracking for the bituminous mixture as well as enhancing its durability.

### 3.2. Microscopic Freeze–Thaw Damage Mechanism of Asphalt Mixture Based on CT Images

#### 3.2.1. Evolution Law of Void Content and Void Number

The content as well as the number of voids in the bituminous mixtures are two basic indicators to describe the characteristics of voids, which may be utilized for analyzing voids’ evolution during freezing and thawing cycling damage.

- Void Content

**Figure 6:**
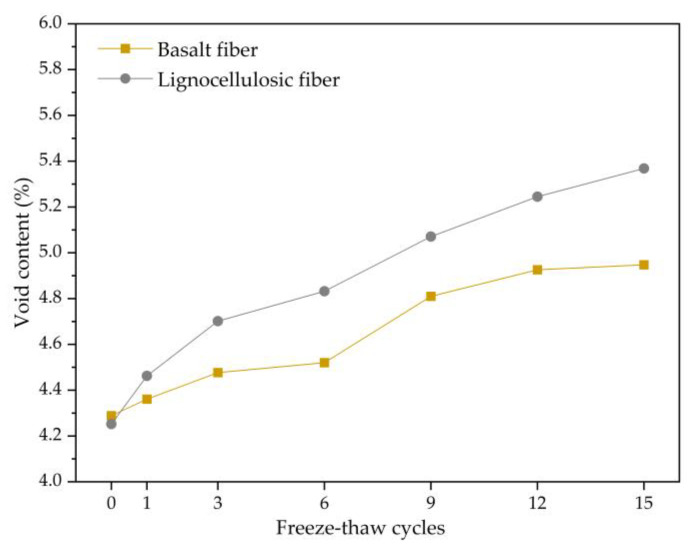
Change trend of void content.

The calculation of the void content for these two bituminous concretes during the freezing and thawing repeated process is performed by calculating the percentage of pixels number inside the void boundaries to the total pixel number in the cross-section. The void content changes vary for the freezing and thawing number, as plotted in Figure 6. Through analysis, the void content of two bituminous concretes follows a similar trend as the experimentally measured void content, increasing with the freezing and thawing repeated process continuing. A statistical analysis of the experimental outcomes shows that scanned void content is about 20% higher than the experimental void content.

- Void Number

The void number counted here is obtained by threshold segmentation of voids in the scanned image. The voids in the image are averaged and weighted along the height direction of the specimen, which is computed:

(1) $$N_{v}=\sum_{i=1}^{n}N_{vi}/n,$$

*N_v_* is the average void number in each layer of the asphalt mixture, *N_vi_* is the void number in a single-layer CT image, and *n* is the number of scanned layers of the asphalt mixture.

**Figure 7:**
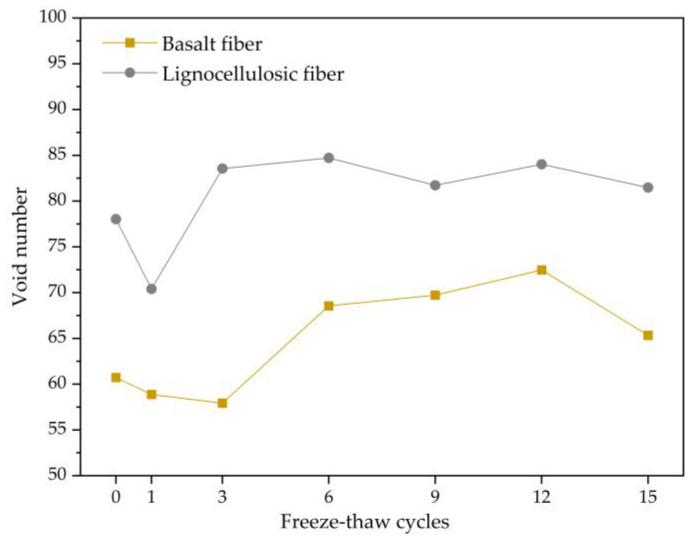
Change trend of void number.

The void number in the two bituminous concretes during the freezing and thawing repeated process was statistically analyzed, and the variation trend for the void number with the freezing and thawing repeated process is plotted in Figure 7. Through analysis, it can be concluded that the variation trend of the void number for two bituminous mixtures is similar to that of the freezing and thawing repeated process continuing. They both decrease and then increase in the early period for the freezing and thawing repeated process, with a large range of variation. As the freezing and thawing repeated process progresses, the void number gradually stabilizes. This is because the fusion of voids is the primary cause influencing the void number during the early freeze–thaw period. Numerous adjacent voids merge under the freezing-thawing action, leading to a decrement in the void number. Afterwards, voids that are greatly affected by freeze–thaw have already mostly merged, and new voids generation becomes the main influencing factor, resulting in an increase in the void number. With the progress of the freezing and thawing cycle tests, a dynamic equilibrium between new voids and merging voids is reached, and the number of voids tends to stabilize. The void number inside the basalt-fiber-modified bituminous mixture is lower in each freeze–thaw cycle period in comparison to the lignocellulosic-fiber-modified bituminous mixture, indicating that the addition of basalt fiber would diminish the void number.

Combining the void number, void content, and mechanical properties, it can be observed that the void number and void content both undergo significant changes in the early period of the freezing and thawing repeated process, indicating that structural failure is a main factor affecting the mechanical properties. As the freezing and thawing repeated process progresses, the changes in both gradually become milder, indicating that inner structural damage is basically complete for the bituminous mixture. The main factor affecting the mechanical properties shifts from internal structural changes to material deterioration.

#### 3.2.2. Evolution Law of the Fractal Dimension

The concept of fractals was first introduced by Mandelbrot in 1975. The fractal dimension can be understood as a measure of the extent to which fractal geometry fills space, characterizing the disorder of sets from the perspective of measure theory and symmetry theory. It is a characteristic quantity for describing complex objects. The calculation method used is the box counting method in fractal theory [37]. This involves dividing an image into square units with a side length of r, recording the minimum number of boxes that contain squares in the image, gradually reducing the size of the units, and obtaining new square box numbers N. Finally, the relationship between the square box number N and the unit side length r can be obtained. If this relationship is a straight line on a logarithmic scale, the negative slope represents the fractal dimension. The fractal dimension can be divided into two types: the contour fractal dimension and the area fractal dimension. This study used the contour fractal dimension. The shape characteristics of voids are mainly manifested in their boundaries. The fractal dimension can effectively describe the irregularity and complexity of boundary curves, quantitatively representing them as a numerical value, which is highly effective for analyzing the shape characteristics of voids.

**Figure 8:**
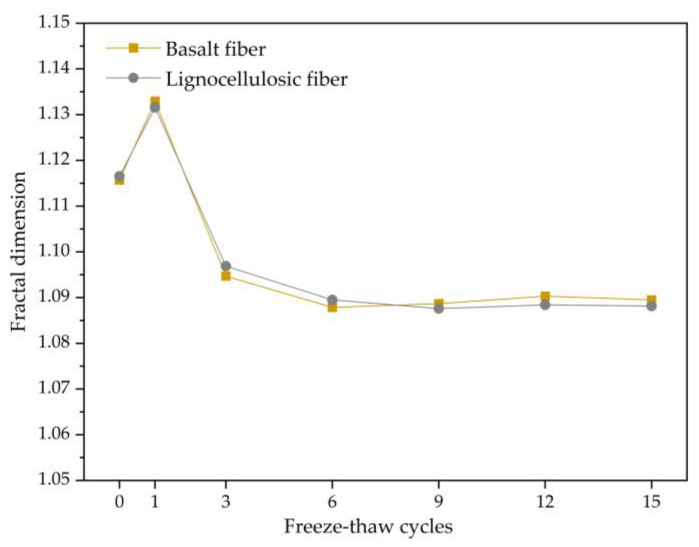
Change trend of fractal dimension.

After calculating the statistics of the void fractal dimensions of the two asphalt mixtures under the freezing and thawing repeated process, the fractal dimension changes with the freezing and thawing repeated process, as shown in Figure 8. From the analysis of Figure 8, it can be observed that the trends of fractal dimension changes for the two asphalt mixtures are the same, which initially increase and then decrease before gradually stabilizing. This is because during the early freeze–thaw period, the void structure undergoes significant changes, and the voids and the outer surface of the aggregate experience peeling off, leading to an increase in the fractal dimension. Afterward, the morphological changes of the voids decrease, and the complexity of the void boundaries decreases, resulting in a decrease in the fractal dimension. As the freezing and thawing repeated process continues, the fractal dimension of the voids tends to stabilize.

#### 3.2.3. Evolution Law of Angularity

The angularity of voids represents the angle changes on the void contour, with larger angle changes indicating greater amplitude of depressions and protrusions on the void contour. As a parameter to describe angularity, it should possess the following attributes: independence from the target size, insensitivity towards the target, and sensitivity towards changes in the target shape. In this study, perimeter-based angularity was adopted, and the quantification indicator was derived from the contrast between voids and equivalent ellipses. The equivalent ellipse retains the shape characteristics of the void contour while attenuating the influence of the contour shape on the angularity quantification. The formula for calculating void angularity is as follows:

*PI* = *P*/*P_e_*, (2)

*PI* is the angularity indicator of the voids, *P* is the perimeter of the void contour, and *P_e_* is the equivalent elliptical circumference of the void.

**Figure 9:**
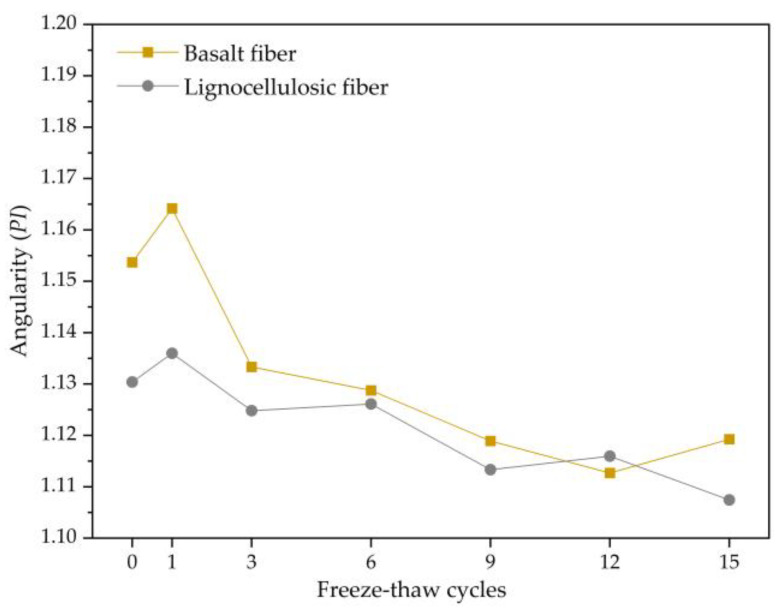
Change trend of angularity.

From Figure 9, it can be observed that the overall trend of angularity changes in the two asphalt mixtures is the same. It increases initially and then decreases with the freezing and thawing repeated process continuing. This is because the morphology of the voids undergoes significant changes during the initial period in the freezing and thawing repeated process, resulting in increased amplitude of depressions and protrusions on the void contour and subsequently increasing the angularity of the voids. As the freezing and thawing repeated process continues, fine aggregates on the void surface, which are more prone to stripping, have been washed away by water, leading to the stabilization of the void angularity.

#### 3.2.4. Evolution Law of Roundness

The roundness is one of the shape characteristics of voids, which represents the degree to which the void contour deviates from a circle. The larger the roundness, the greater the deviation of the void contour from a circle, and the larger the surface angles. The formula for calculating roundness is as follows:

*R* = *L*^2^/4π*A*, (3)

*R* is roundness of the void, *L* is the perimeter of the void contour, and *A* is the void area.

**Figure 10:**
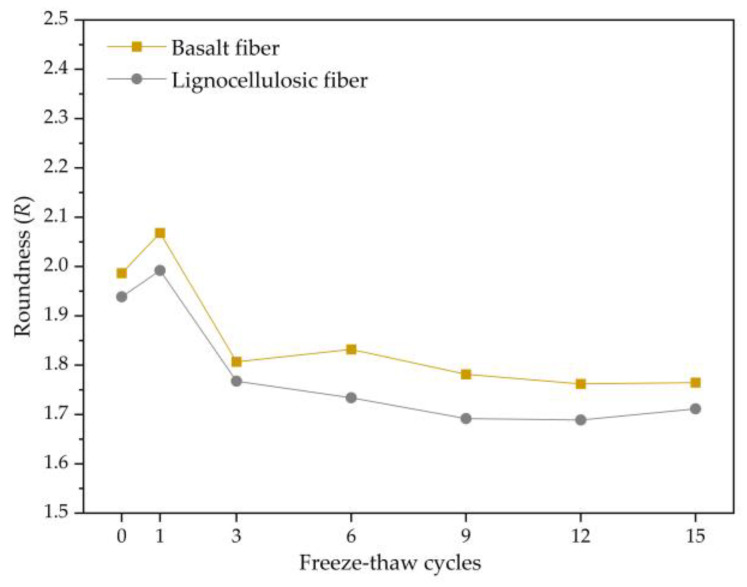
Change trend of roundness.

From the analysis in Figure 10, it can be seen that the roundness trends of the two bituminous mixtures are the same, with both increasing and then decreasing and eventually stabilizing. This is because during the early freeze–thaw period, the void structure undergoes significant deformation, with some adjacent voids experiencing expansion and merging, resulting in an increase in roundness. During freezing, the water in the voids freezes and exerts pressure on the voids. The protruding parts of the voids undergo stress concentration under the pressure, leading to the deformation of the voids and a gradual regularization of the void contour, resulting in a decrease in roundness and approaching a circular shape. After that, the inner structure gradually stabilizes, and the roundness of the voids remains stable. By analyzing the data, it is found that voids with a roundness of less than two account for 75.3% of the total number of voids. Among them, voids with larger roundness mainly come from two sources, one being outer voids in the cross-sectional images, and the other being small voids.

#### 3.2.5. Evolution Law of the Aspect Ratio

The aspect ratio is also one of the shape characteristics of voids, which is the ratio of the major axis to the minor axis for the equivalent ellipse of the void, representing the degree to which the void’s equivalent ellipse approximates a circle. The formula for calculating the aspect ratio is as follows:

*AR* = *R_l_*/*R_s_*, (4)

*AR* is the ratio of the length of the void to the length of the axis, *R_l_* is the major axis of the void’s equivalent ellipse, and *R_s_* is the short axis of the void’s equivalent ellipse.

**Figure 11:**
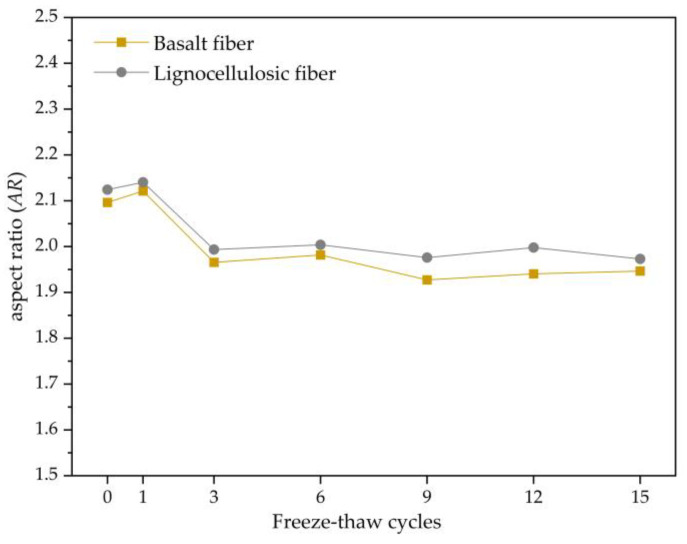
Change trend of aspect ratio.

From the analysis in Figure 11, the aspect ratio trends of these two asphalt mixtures are the same, with both increasing and then decreasing and eventually stabilizing. This is because during the early period of freeze–thaw, the void structure undergoes significant changes, with some adjacent voids merging into interconnected voids, resulting in an increase in the aspect ratio. During the freezing period, the water in the voids freezes and exerts pressure on the voids, gradually making the void shape regular and decreasing the aspect ratio. With the freezing and thawing repeated process continuing, the internal structure of these asphalt mixtures gradually stabilizes, and the aspect ratio of the voids also stabilizes.

## 4. Conclusions

In this study, the freeze–thaw damage mechanism of SBS bituminous mixtures containing basalt fiber and lignocellulosic fiber was analyzed based on the microscopic void characteristics. The objective was to understand how the presence of these reinforcement fibers affects the microstructure and void characteristics of bituminous mixtures during the freezing and thawing repeated process. The experimental results revealed several important findings.

(1) The mechanical performance of two types of asphalt mixtures decreases with an increasing number of freeze–thaw cycles. The rate of decrease is faster in the early stages of the freeze–thaw cycles and gradually becomes more moderate as the freeze–thaw tests continue. The air void content and mechanical properties of basalt-fiber-modified SMA-13 are higher than those of lignocellulosic-fiber-modified SMA-13, indicating that adding basalt fibers to SMA-13 asphalt mixture can improve its mechanical performance.
(2) Both types of asphalt mixtures exhibit an increasing trend in damage degree with an increasing number of freeze–thaw cycles, indicating that the damage is irreversible. The stability damage degrees of the two asphalt mixtures are similar, but the splitting strength damage degree and stiffness modulus damage degree of basalt-fiber-modified SMA-13 are lower than those of lignocellulosic-fiber-modified SMA-13. This also suggests that adding basalt fibers to SMA-13 can enhance its resistance to freeze–thaw damage.
(3) Under the repeated action of freeze–thaw cycles, the surface wear of asphalt mixtures is a complex dynamic process. Revealing the mechanism of surface wear of the mixture using fractal theory and describing the surface wear behavior and void deformation characteristics of the mixture using the fractal dimension, angularity, roundness, and aspect ratio are highly reasonable and effective approaches.
(4) The variation law of the fractal dimension, angularity, and roundness is consistent, indicating that the effect of freeze–thaw on the destruction of voids is concentrated in the early stage, and the effect is significant.

The findings from this study have important implications for the design and construction of asphalt pavements. By incorporating basalt fiber and lignocellulosic fiber into SBS asphalt mixtures, engineers can enhance the durability and the resistance to freeze–thaw damage, leading to longer service life and reduced maintenance costs. This finding can be useful for engineers and designers involved in asphalt pavement design, as it provides a potential solution to the problem of freeze–thaw damage in asphalt pavements. The microscopic void characteristics analysis provides a valuable understanding of the intricate mechanisms underlying the freeze–thaw damage processes, allowing for the development of more effective mitigation strategies. In future work, the long-term durability beyond 15 freeze–thaw cycles should be carried out for SBS asphalt mixtures, and the mechanisms explaining how the fibers influence void characteristics should be explored in depth based on the analysis of fiber–matrix interactions considering fiber parameters.

## Figures and Tables

**Table 1:** The technical parameters of SBS type I-C.

Technical Parameter		Unit	Requirement	Result
Penetration (25 °C, 100 g, 5 s)		0.1 mm	60~80	71
Ductility (5 °C, 5 cm/min)		cm	≥30	45
Softening point		°C	≥55	60.5
Density		g/cm^3^	-	1.018
Flash point		°C	≥230	262
Elastic recovery (25 °C)		%	≥65	88
RTFOT	Mass variation	%	≤±1.0	−0.094
	Penetration ratio	%	≥60	66.9
	Ductility	cm	≥20	33.2

**Table 2:** Technical indicators for coarse aggregate quality.

Technical Indicators		Requirement	Result
Abrasion loss (%)		≤28	17.9
Crushing value (%)		≤26	13.6
Apparent relative density	13.2 mm	≥2.6	2.836
	9.5 mm		2.805
	4.75 mm		2.726
Water absorption rate (%)	13.2 mm	≤2.0	0.6
	9.5 mm		0.28
	4.75 mm		0.7
Robustness (%)		≤12	5
Needle and flake content (%)		≤15	9.2
Particle content < 0.075 (%)		≤1	0.3
Soft stone content (%)		≤3	1

**Table 3:** Technical indicators for fine aggregate quality.

Technical Indicators	Requirement	Result
Apparent relative density	≥2.5	2.723
Water absorption rate (%)	-	0.64
Angularity (s)	≥30	39.9
Sand equivalent (%)	≥60	68

**Table 4:** Technical indicators for mineral powder quality.

Technical Indicators		Requirement	Result
Moisture content (%)		≤1	0.3
Particle size range (%)	<0.6 mm	100	100
	<0.15 mm	90~100	92.5
	<0.075 mm	75~100	81.8
Apparent density (t/m^3^)		≥2.5	2.712
Hydrophilicity coefficient		<1	0.63
Heating stability		Actual record	Good, no color change
Plasticity index (%)		<4	2

**Table 5:** Performance indicators of basalt fiber and lignocellulosic fiber.

Technical Parameter	Unit	Lignocellulosic Fiber	Basalt Fiber
Fiber length	mm	1.1	6
Specific gravity	-	0.91	2.6
Color	-	Grayish white	Dark brown
Acid and alkali corrosion resistance	-	Good	Good
Tensile strength	MPa	300	3200
Elastic modulus	GPa	35	95
Elongation	%	2.5	3.2

**Table 6:** Volume index of asphalt mixture.

Mixture Type	Asphalt-to-Aggregate Ratio	Void Content (%)	VMA (%)	VFA (%)
Basalt-fiber- modified SMA-13	5.7	3.8	16.6	77.1
	5.9	3.6	16.8	78.6
	6.1	3.3	16.9	80.5
Lignocellulosic-fiber- modified SMA-13	6.1	3.8	17.2	77.9
	6.3	3.5	17.3	79.8
	6.5	3.2	17.2	81.4

## Data Availability

Not applicable.
